# Sternal reentry in a patient with previous deep sternal wound infection managed with horizontal titanium plate fixation

**DOI:** 10.1186/1749-8090-5-56

**Published:** 2010-07-22

**Authors:** Richard Baillot, Éric Dumont, Pierre Voisine

**Affiliations:** 1 Department of Cardiac Surgery, Laval University, Quebec, Canada

## Abstract

Redo open-heart surgery and sternal reentry in patients with previous deep sternal wound infection (DSWI) and absence of sternal integrity can be a delicate and morbid task due the lack of a dissection plane between the heart and the surrounding soft tissues. Delayed sternal reconstruction and osteosynthesis with horizontal titanium plating fixation (Synthes) following vacuum assisted therapy (KCI) has recently been proposed and adopted for the treatment of DSWI. We present such a case of a patient who was successfully reoperated for valve replacement three years after coronary artery bypass grafting complicated by DSWI and initially treated with titanium plate fixation.

## Introduction

Deep sternal wound infection remains a feared complication of cardiac surgery still associated with significant morbidity and mortality. Furthermore, there is a lack of consensus for its definitive management [1,2]. We have recently adopted the routine use of negative wound pressure therapy (VAC -KCI) [3-5] after initial wound debridement as a bridge to delayed chest wall reconstruction with horizontal titanium plate fixation (Synthes) [6]. This report will focus on the case history of a patient managed according to this approach and who eventually required a redo open heart surgery.

## Case report

**Figure 1:**
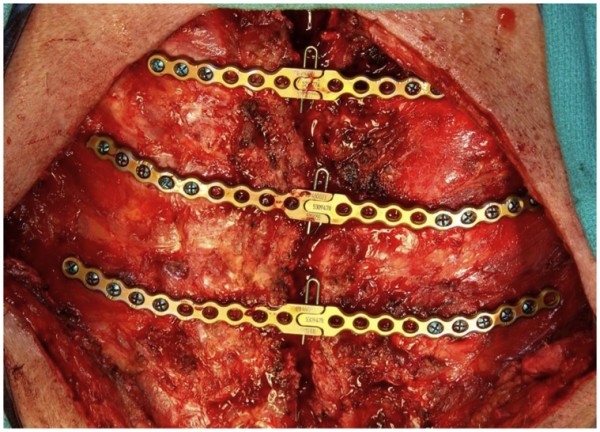
**Horizontal titanium plate fixation**.

A 73--year old active patient underwent triple coronary artery bypass grafting (CABG) in 2005 at an outside hospital. This initially uneventful procedure was followed by a *S.Aureus *DSWI which was referred to our center and managed with aggressive debridement, VAC therapy and then delayed sternal wound reconstruction with horizontal titanium plate fixation (Figure 1) and pectoralis myocutaneous flaps.

Three years later the patient was investigated following complaints of recurrent chest pain and shortness of breath. A repeat coronary angiogram showed that the two vein grafts were occluded while the left internal thoracic artery directed to the left anterior descending coronary artery remained patent and functional. Severe aortic stenosis was documented by echocardiography with an aortic valvular area of 0.6 cm2 and respective maximal and mean gradients of 59 and 38 mmHg. A transapical aortic valve replacement and percutaneous coronary dilatation were initially considered but a formal midline sternotomy finally chosen, as it was felt he was a good candidate for conventional surgery.

**Figure 2:**
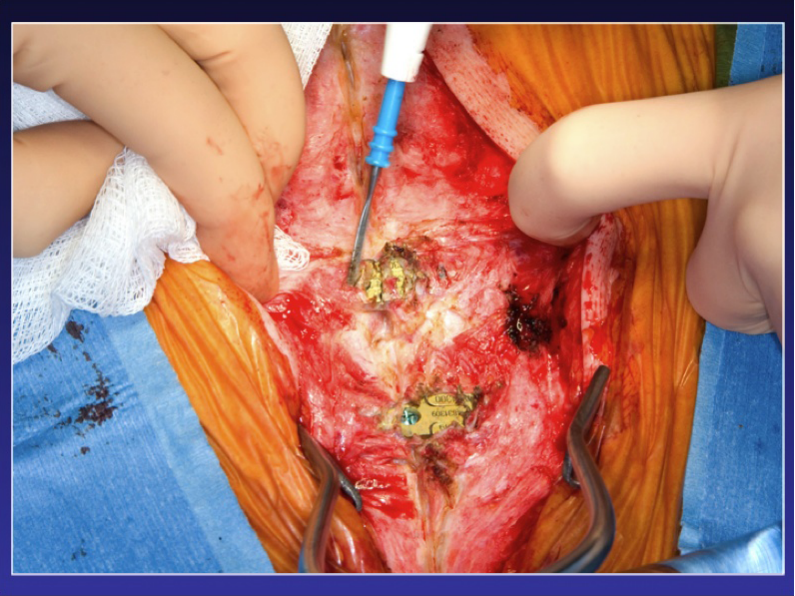
**Redo sternotomy with plate exposure**.

**Figure 3:**
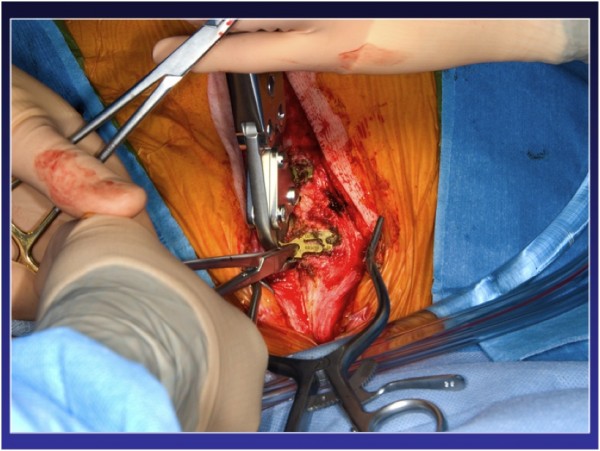
**Titanium plate cut in the middle**.

**Figure 4:**
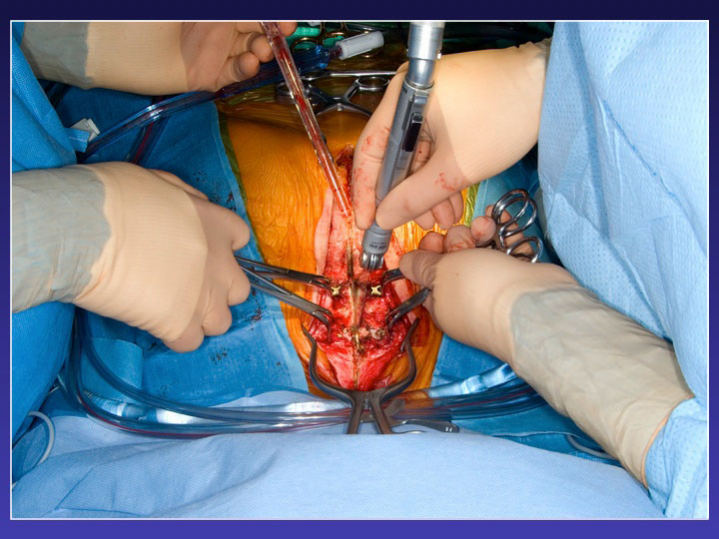
**Titanium plate pull upward to help redo stenotomy with the oscillation saw**.

After removing the locking pin and cutting the titanium plates along the sternal midline, sternal re-entry could be performed uneventfully with the oscillating saw while the plates were pulled upward (Figure 2, Figure 3, Figure 4). The plates can be completely removed, but this requires extensive debridement under the myocutaneous flaps, which was felt to be an avoidable cause of potential complications by simply leaving the plates in place. The sternal bone was densely ossified and bony union complete without unusual substernal scarring, and a decision was made to proceed to primary closure using steel wires. Otherwise the plates can be re-approximated by inserting a new locking pin in the closure mechanism, if sufficient care is exerted at preserving its integrity at the time of sternal retractor placement. An aortic valve replacement (Magna 23 mm) with a triple CABG (Saphenous vein graft to the left anterior descending artery and sequential saphenous vein grafting to the first obtuse marginal and a postero-lateral branch) had to be done due to an unfortunate avulsion of the left ITA during the dissection of the lateral aspect of the ventricle. At the time of the avulsion, he was fully cannulated and never suffered any ischemia. The procedure as well as the early post-operative period were uncomplicated and the patient was discharged on post-op day five with normal ventricular function and a mean trans-aortic gradient of 12 mmHg and an effective valvular area of 1.8 cm2.

## Discussion

Deep sternal wound infection will remain a costly and worrisome complication of cardiac surgery despite better care of septic patients and management with muscle flaps. Sternal preservation with osteosynthesis has previously been suggested to make cardiac reoperation a safer procedure[7]. However, literature on the subject remains scarce, and to our knowledge this is the first paper to report such a surgery in a patient previously managed with horizontal titanium fixation following DSWI. Sternal plating is associated with better and faster osseous healing both experimentally[8] and clinically and it has even been suggested in some instances for primary operations, with the objective of reducing the risk of sternal instability often associated with DSWI in patients presenting with significant risks factors of dehiscence such as obesity and diabetes[9]

Since 2002, we have adopted the routine use of negative pressure wound therapy after debridement in patients presenting with DSWI in our center, and have been able to better preserve sternal integrity with this approach. It usually takes 2 weeks following initial and repeat debridements to obtain negative wound cultures in these cases and then a full sternal reconstruction can then be performed with titanium plates covered with pectoralis myocutaneous flaps even after partial bone losses from the manubrium or each hemi-sternum.

With increasing life expectancy in industrialized countries, reoperation is a likely possibility in open heart surgery patients often presenting with degenerative valvular pathologies such as aortic stenosis. The volume of transapical aortic valvular replacement has been rapidly increasing worldwide and will be an option in these patients but this procedure is also carrying its own risk and at this point in time is still offered to patients who are not good surgical candidates[10]. If the patient is in good condition, reoperation is feasible and sternal reentry can be done safely and with minimal risk even after sternal reconstruction with horizontal titanium plating.

## Abbreviations

CABG: Coronary artery bypass grafting; VAC: Vacuum Assisted Therapy/Negative pressure wound therapy

## Competing interests

The authors declare that they have no competing interests.

## Authors' contributions

All authors read and approved the final manuscript.

## Consent

Data from all patients operated at the IUCPQ are entered in the surgical data base without patient's name and according to current ethical rules of the research center.
